# 经典型骨髓增殖性肿瘤伴高血压的临床研究进展

**DOI:** 10.3760/cma.j.cn121090-20240811-00300

**Published:** 2025-06

**Authors:** 文豪 张, 古生 唐

**Affiliations:** 海军军医大学第一附属医院血液病科，上海 200433 Department of Hematology, the First Affiliated Hospital of Naval Medical University, Shanghai 200433, China

## Abstract

慢性骨髓增殖性肿瘤（MPN）是一类以造血干细胞克隆性增生为特征的获得性恶性肿瘤，其中真性红细胞增多症、原发性血小板增多症和原发性骨髓纤维化被称为经典型费城染色体阴性的MPN。高血压是MPN患者最常见的伴发疾病，对MPN的临床表现、病情进展、治疗及预后有显著影响，但在临床工作中可能并未引起足够重视。本文对MPN伴高血压的流行病学及临床问题进行综述，以协助临床医师对该类患者进行个体化治疗。

## Abstract (en)

Chronic myeloproliferative neoplasms（MPNs）are a group of acquired malignant tumors characterized by clonal proliferation of hematopoietic stem cells, among which polycythemia vera, essential thrombocythemia, and primary myelofibrosis are known as the classical Philadelphia chromosome-negative MPN. Hypertension is the most common complication in patients with MPN and has a significant effect on the clinical presentation, disease progression, treatment, and prognosis of patients with MPN. However, it may not have received sufficient attention from hematologists in clinical practice. This article reviews the epidemiology and clinical issues of patients with MPN and comorbid hypertension to assist clinicians in providing individualized treatment for these patients.

费城染色体阴性（Ph^-^）骨髓增殖性肿瘤（MPN）是一组以造血干细胞克隆性增生为特征的获得性恶性肿瘤。经典型Ph^-^ MPN主要包括真性红细胞增多症（PV）、原发性血小板增多症（ET）和原发性骨髓纤维化（PMF）。由于血小板、红细胞和白细胞计数的增加和功能异常在Ph^-^ MPN患者中常见，心脑血管事件是此类患者最常见的并发症。与此同时，MPN患者大部分为中老年人（ET患者的中位发病年龄为55岁，PV为64岁，PMF为63岁），而高血压、糖尿病、高脂血症等多种心血管危险因素也更多地出现在中老年人群中[1]，这些危险因素进一步增加了MPN患者心脑血管事件的风险。高血压是成人人群中最常见的心血管危险因素[2]。MPN伴高血压患者的临床表现和结局具有一定的特征，但缺乏文献总结。本文拟对此类患者的流行病学及高血压对MPN病程的影响进行综述，希望有助于对该类患者制定个体化管理和治疗措施，减少心血管事件的发生。

1. Ph^-^ MPN患者伴高血压机制：国际高血压学会（ISH）2020年指南提出，连续测量2～3次诊室血压≥140/90 mmHg（1 mmHg＝0.133 kPa）即可诊断为高血压，目前我国高血压的诊断也采用此标准。美国2017年将高血压标准定为高于130/80 mmHg。多项大数据研究分析表明，高血压是MPN患者最常见的并发症。一项研究统计了美国退伍军人健康管理局中诊断为PV的7 718例患者，显示高血压是PV患者最常见的伴发疾病（71％），超过血脂异常（54％）和糖尿病（24％）[3]。另一项前瞻性观察性临床研究（REVEAL）纳入2 510例PV患者，显示PV患者高血压的发病率高达70.6％[4]。回顾性分析Truven Health MarketScan数据库中2 856例PV患者（其中高危患者1 823例，低危患者1 033例），对两组患者进行比较，最常见的并发症分别是高血压（65.0％对43.1％）、2型糖尿病（21.7％对10.1％）和充血性心力衰竭（6.6％对0.6％）[5]，提示伴发高血压等疾病是心脑血管事件的高危因素。Accurso等[6]回顾性分析了603例MPN患者，约63％的PV、64％的ET、15％的纤维化前期PMF（Pre-PMF）和63％的明显期PMF/继发性PMF（Overt-PMF/SMF）患者存在高血压。来自马来西亚的研究显示，MPN各种亚型中，伴发高血压的频率由高到低依次为PV（58％）、ET（42％）、PMF（41％）、MPN不能分类型（36％）、慢性嗜酸性粒细胞白血病（33％），该研究还提示，高血压可能与JAK2V617F基因突变相关[7]。巴基斯坦的一项回顾性研究显示，51例PV患者并发高血压的频率高达90.2％[8]。一项意大利的研究主要包含ET患者，显示与其他心血管危险因素（血脂异常24.0％、糖尿病14.2％）相比，高血压（63.5％）仍然是最常见的并发症[9]。

我国2018年老年患者高血压患病率调查显示，60～70岁、70～80岁、≥80岁人群中高血压的患病率分别为54.4％、65.2％、66.7％[10]。甘肃省人民医院血液科的回顾性研究纳入126例MPN患者，并发高血压的发生率分别为PV（50.0％）、ET（49.3％）、PMF（10.5％）[11]；北京大学首钢医院对在本院就诊的179例MPN患者进行脑卒中分析时发现，MPN患者并发高血压的发生率为37.43％，提示高血压是MPN患者发生脑卒中的危险因素[12]。福建医科大学附属医院对176例Ph^-^ MPN患者进行血栓形成分析时发现，MPN患者高血压的发生率为65.34％，单因素Logistic回归分析显示，高血压是Ph^-^ MPN患者血栓事件的危险因素（*OR*＝1.968，*P*<0.05）[13]。

MPN患者的高血压发病率似乎略高于正常人群（43.1％～90.2％，尤其是PV患者），可能与JAK2V617F基因突变相关[7]。JAK2V617F突变通过激活基质金属蛋白酶诱导主动脉弹性层降解[14]。MPN患者存在心血管、神经系统等方面的特殊表现，也可能是发生高血压的原因之一。此外，MPN患者中普遍出现的血小板计数高及血小板功能障碍可能引起血栓形成和高血压[15]–[16]。一氧化氮（NO）会抑制血管紧张素导致的平滑肌细胞收缩[17]，部分MPN药物会抑制JAK2表达，从而减弱Raf-1/MEK1/Sp-1信号及内皮型一氧化氮合酶（eNOS）的表达，使血压升高[18]。但目前对于MPN或MPN药物引起高血压的观点均为间接推断或猜测，缺乏实质性的直接证据，仍需要进一步探索。

2. MPN伴高血压患者的特殊临床表现：Patino-Alonso等[19]将MPN患者与健康受试者按照2∶1的比例匹配后分析发现，MPN患者的舒张压和收缩压与健康受试者并无差别。然而，Akdi等[20]的研究发现，尽管PV患者的日间收缩压、舒张压及平均血压与健康人并无明显差别，但其表现出非勺型的昼夜血压节律，PV患者的夜间收缩压及舒张压均升高，且和HGB、红细胞比容相关。波兰的一项病例对照研究同样未发现PV患者与健康对照组在诊室血压或24 h收缩压、舒张压，心率或抗高血压药物使用方面存在显著差异。PV患者超声心动图室间隔和侧壁的收缩速度与整体纵向应变、整体圆周应变和整体径向应变均下降，而等容舒张时间增加[21]。

伴高血压的MPN患者在心血管、神经系统及肾脏、视网膜等微血管丰富器官可能有多种特异性表现，如出现视网膜中央动脉闭塞，并表现为突发失明。可能通过肾硬化加重MPN相关肾小球病，并出现极性血管扩张和渗出性病变[22]–[23]。比较PV伴高血压患者与对照组（无PV，但性别、年龄、血压和药物使用数量与试验组匹配的高血压患者）的交感神经系统活动及视网膜和肾脏的灌注情况，显示PV患者24 h心率和神经肌电活动均较低（特别是白天），并且显示PV患者的血浆游离肾上腺素和醛固酮浓度较高，视网膜灌注减少（与PV患者的红细胞计数呈负相关）以及肾动脉阻力增高[24]–[25]。德国的MPN生物登记研究组纳入1 420例患者（ET、PV、PMF分别占33.7％、38.5％、27.9％），伴高血压者占49％，根据估算肾小球滤过率（eGFR）将其分为3组，Logistic分析显示，高血压是MPN患者出现肾功能不全的重要危险因素[26]。

3. MPN伴高血压患者的血栓形成情况：血栓栓塞是目前临床中影响MPN患者无事件生存期和总生存期最常见、最重要的危险因素，病死率占MPN总病死率的35％～70％[27]。目前认为，高血压会增加MPN患者的血栓形成风险，但部分研究结论具有一定争议。

与ET相比，高血压在PV患者中更普遍，血压与红细胞比容呈正相关，高血压使低风险PV患者血栓形成的发生率升高且无血栓生存率下降[28]。一项国际合作研究对891例ET患者进行6年的随访观察，显示发生血栓事件的109例（79例动脉血栓，37例静脉血栓）患者中，高血压是动脉血栓形成的预测因素[29]。对105例ET患者和62例PMF患者的血栓事件进行回顾性分析，显示高血压是动脉血栓形成的危险因素，但对ET患者的静脉血栓形成没有影响[30]。对128例随访期间发生血栓的女性ET患者进行单变量分析，发现高血压是影响血栓形成的重要因素（未区分动、静脉血栓）[31]。欧洲合作组织分析了接受低剂量阿司匹林治疗的1 638例PV患者，认为高血压并不是影响治疗中患者动静脉血栓形成的主要因素[32]。

匈牙利的一项研究对101例住院ET患者进行血栓形成分析，显示尽管高血压是患者最常见的并发症，但血栓的形成与高血压并无相关性（未区分动、静脉血栓）[33]。回顾性分析132例ET患者的心血管危险因素（吸烟32例，高血压27例，高胆固醇血症21例，糖尿病4例）对血栓形成的影响，对其中发生血栓的59例患者进行多因素分析（53例患者发生98次动脉血栓，15例患者发生27次静脉血栓），发现吸烟是血栓形成的唯一独立危险因素，高血压不是独立危险因素[34]。Horvat等[35]将258例MPN患者按照亚型分为PV（70例）、ET（134例）和PMF（54例）三组，分析血栓形成类型与心血管危险因素的相关性，结果表明，在PV和PMF组中，高血压合并至少一种心血管危险因素是动脉血栓形成的危险因素，而在ET患者中则同时为动静脉血栓形成的危险因素。上述研究提示，除高血压和MPN亚型因素外，是否存在其他心血管危险因素及其数量和类型的差异可能是不同研究高血压与血栓相关性差异的重要影响因素。

4. MPN伴高血压患者的治疗选择：当前PV患者高血压的首选药物推荐血管紧张素转换酶抑制剂（ACEI）。借鉴肾移植后红细胞增多症患者应用ACEI控制高原缺氧患者血压的随机前瞻性实验[36]–[37]，Barbui等[38]回顾了ECHANISM数据库中1 638例伴高血压的PV患者的临床数据，61％的患者进行降细胞治疗，大多数病例采用羟基脲，其中647例接受了抗高血压药物单药或联合治疗，应用的药物依次是ACEI（285例）、钙通道阻滞剂（177例）、β受体阻滞剂（103例）、利尿剂（96例）。服用ACEI的患者同其他组相比，性别、年龄、常规风险分层、血细胞计数和除高血压外的心血管风险因素以外的其他因素之间并无显著差异，但ACEI组患者接受化疗的频率显著低于应用其他药物的患者（*OR*＝0.78，*P*＝5.006），多变量分析也证实了这一点（*OR*＝0.62，95％ *CI* 0.44～0.88，*P*＝0.008）。而且尽管ACEI组PV患者使用的化疗药物更少，但两组血细胞比容的中位数没有差异[38]，可能与PV患者骨髓局部血管紧张素转换酶和其他血管紧张素组成成分合成增加相关[39]。另外，ACEI对PV患者的肾功能也有积极影响，对75例应用ACEI的PV患者进行回顾性研究，发现25例患者第12个月eGFR升高超过10％（*OR*＝6.97，95％ *CI* 3.83～8.89，*P*＝0.008）[40]。鉴于MPN相关高血压的处理具有重要临床意义，目前也无统一的共识和建议，我们尝试就相关问题进行荟萃分析，但PubMed、Embase和cochrane等数据库中仅有4篇文献符合筛选标准，均为临床研究中的队列研究或病例对照研究。评估后发现这4篇文章中使用不同结局指标进行评估，文献异质性过高，并不能进行合并分析[4],[38],[40]–[41]。因此，对于伴高血压的PV患者应用ACEI的临床研究尚缺乏进行荟萃分析的基础，期待有循证医学证据支持的新研究出现。

实际临床工作中，对MPN的治疗也可能会影响高血压病程的进展，导致降压药的使用量或种类有明显改变。临床工作中发现许多MPN患者，尤其是PV患者在放血或红细胞单采治疗后、使用羟基脲或干扰素降细胞治疗后，血压出现明显好转，治疗用药减少，甚至停药。推测其机制可能与降细胞治疗后循环血细胞数量显著减少，血液淤滞减轻，血流阻力减少相关。该临床现象提示，在治疗MPN的同时，需要关注并提醒患者对血压进行动态监控，以早期发现血压波动并进行药物调整，避免低血压事件的发生。但对于MPN治疗影响高血压病程进展的相关研究报道目前未检索到，需要进一步临床研究数据证实。此外，尽管比例不高，但也有治疗MPN的药物引起高血压的报道。一项随机Ⅲ期临床试验纳入PV和ET患者，显示羟基脲和干扰素引发的3～4级不良反应中高血压分别占5％和13％[42]。一项为期5年的随机3b研究将149例PV患者分为芦可替尼组（74例）与最佳治疗组（羟基脲38例、干扰素或聚乙二醇干扰素9例、哌泊溴烷5例、来那度胺1例、随诊22例），两组最常见的3～4级不良事件均为高血压（8例对3例）[43]。2020年一项3b阶段的多中心随机、开放标签、非劣势研究评估了146例ET受试者服用阿那格雷和羟基脲的安全性，研究登记的紧急不良事件中，高血压是最常见的心血管疾病［阿那格雷：9例（11.8％）；羟基脲：1例（1.4％）］[44]。尽管原因尚不清楚，但上述研究提示，在MPN患者的治疗和随访过程中需要提醒患者注意血压监测，必要时需专科随访处理。

高血压是MPN患者最常见的伴发疾病。对MPN患者预后最重要的血栓事件研究中，大部分观点认为高血压可增加动脉血栓事件的发生率，但高血压与静脉血栓的相关性不大。MPN患者并发高血压和其他一个以上心血管因素时更容易发生血栓事件。伴高血压的MPN患者表现出的部分特殊临床表现中，对肾脏、视网膜等微血管丰富器官的危害值得我们警惕，与患者的预后和生活质量息息相关。针对伴高血压MPN患者治疗的特定研究较少，除PV患者首选ACEI有明确的证据外，其他MPN亚型的高血压发生率也较高，但相关治疗缺乏系统的总结。治疗MPN的药物引起药物性高血压的情况相对少见，但在日常的临床工作中也应关注。MPN治疗本身对高血压进展的影响值得临床进一步研究。
